# Correction: A Reciprocal Model of Face Recognition and Autistic Traits: Evidence from an Individual Differences Perspective

**DOI:** 10.1371/journal.pone.0105669

**Published:** 2014-08-11

**Authors:** The PLOS ONE Staff

The third author’s name is spelled incorrectly. The correct name is: K. Suzanne Sherf. The correct citation is: Halliday DWR, MacDonald SWS, Sherf KS, Tanaka JW (2014) A Reciprocal Model of Face Recognition and Autistic Traits: Evidence from an Individual Differences Perspective. PLoS ONE 9(5): e94013. doi:10.1371/journal.pone.0094013
